# Homocysteine induces glyceraldehyde-3-phosphate dehydrogenase acetylation and apoptosis in the neuroblastoma cell line Neuro2a

**DOI:** 10.1590/1414-431X20154543

**Published:** 2016-01-19

**Authors:** M. Fang, A. Jin, Y. Zhao, X. Liu

**Affiliations:** Department of Neurology, Shanghai Tenth People’s Hospital, Shanghai, China

**Keywords:** Homocysteine, Glyceraldehyde-3-phosphate dehydrogenase, Apoptosis, Acetylate, p300/CBP

## Abstract

High plasma levels of homocysteine (Hcy) promote the progression of neurodegenerative diseases. However, the mechanism by which Hcy mediates neurotoxicity has not been elucidated. We observed that upon incubation with Hcy, the viability of a neuroblastoma cell line Neuro2a declined in a dose-dependent manner, and apoptosis was induced within 48 h. The median effective concentration (EC_50_) of Hcy was approximately 5 mM. Glyceraldehyde-3-phosphate dehydrogenase (GAPDH) nuclear translocation and acylation has been implicated in the regulation of apoptosis. We found that nuclear translocation and acetylation of GAPDH increased in the presence of 5 mM Hcy and that higher levels of acetyltransferase p300/CBP were detected in Neuro2a cells. These findings implicate the involvement of GAPDH in the mechanism whereby Hcy induces apoptosis in neurons. This study highlights a potentially important pathway in neurodegenerative disorders, and a novel target pathway for neuroprotective therapy.

## Introduction

Homocysteine (Hcy), a non-essential sulfur-containing amino acid derived from methionine metabolism, has been implicated in the development and progression of neurodegenerative diseases (1 2 3-4). Hcy catabolism depends on folate, vitamin B6 and B12 (1). Decreased activity of these enzymes or folate, or B vitamin deficiency, can lead to elevated levels of Hcy (hyperhomocysteinemia, HHcy) (5).

HHcy is an independent risk factor for vascular diseases, and is associated with the progression of neurodegenerative disorders, such as Alzheimer’s disease and Parkinson’s disease (1-4). Hcy is a neurotoxic agent that directly injures neurons (6,7). However, the mechanism underlying Hcy-induced neural apoptosis is not yet well understood. Previous studies have revealed that HHcy reduces DNA methylation, impairs gene transcription and inhibits DNA repair; thus inducing gene mutation and cell apoptosis (7). Hcy also activates cysteine containing specific protease (caspase) and p53 to reduce mitochondrial membrane potential and thus induce cell apoptosis (8). In addition, Hcy induces oxidative damage, activates NMDA receptors, increases the excitotoxicity of glutamic acid, and induces reactive oxygen species (ROS) production (9).

Glyceraldehyde-3-phosphate dehydrogenase (GAPDH) is a glycolytic enzyme with a key role in energy production, catalyzing the conversion of glyceraldehyde-3-phosphate to 1,3-bisphosphoglycerate (10). Recently, additional non-glycolytic functions of GAPDH have been identified (11 12 13 14 15 16-17), including a role in apoptosis, first demonstrated in cultured cerebellar granule cells and cortical neurons undergoing spontaneous apoptosis (18). Previous reports have demonstrated that the overexpression and nuclear accumulation of GAPDH are early, critical events in apoptosis pathways (19). GAPDH has also been implicated in the neuronal cell death observed in neurodegenerative diseases, such as Parkinson’s, Huntington’s, and Alzheimer’s diseases (18,20 21 22 23 24 25-26). Although both GAPDH and Hcy are strongly involved in neurodegenerative diseases, especially neuron cell apoptosis, no study has established a clear link between the two factors in connection with programmed cell death. Therefore, this study aimed to further elucidate the mechanism by which Hcy promotes the progression of neurodegenerative diseases, particularly its effect on GAPDH cellular localization and acetylation, which are critical in apoptosis.

Using the mouse neuroblastoma cell line Neuro2a, we demonstrated that Hcy induces neuronal cell apoptosis. Interestingly, Neuro2a cell treatment with Hcy resulted in increased GAPDH acetylation and higher acetyltransferase p300/CBP levels. These findings clearly indicate the involvement of GAPDH in Hcy induced neuron apoptosis.

## Material and Methods

### Cell culture

Mouse neuroblastoma cell line, Neuro2a (Shanghai Institute of Chinese Academy of Science, China), was cultured in Eagle’s minimal essential medium (MEM), supplemented with 10% fetal bovine serum (FBS, Gibco, USA), 2 mM L-glutamine, 1 mM sodium pyruvate, 100 μg/mL streptomycin and 100 U/mL penicillin, at 37°C in a 5% CO_2_ humidified atmosphere. Cells were subcultured before reaching confluence, with medium renewal every two days.

### Cell viability assay

Cell viability was analyzed using the WST-8 Cell Counting Kit-8 (Dojindo, Japan), a sensitive nonradioactive colorimetric assay for determining the number of viable cells, according to the manufacturer’s protocol. Briefly, Neuro2a cells were seeded onto 96-well plates in MEM supplemented with 10% FBS and Hcy (10, 20, 30, 40 or 50 mM), and incubated for 24 or 48 h before medium was replaced with fresh medium containing 10% CCK-8. Control cells were cultured in the absence of Hcy. After 2 h at 37°C, the absorbance at 450 nm was measured by microplate reader (BioTek Instruments, USA).

### Assessment of apoptosis

Neuro2a cells were incubated in the absence or presence of the previously defined EC50 of Hcy (5 mM) for 24 or 48 h. Apoptotic cells were distinguished by Annexin V-FITC and propidium iodide (PI) staining using a commercially available kit (BD Pharmingen, USA). Briefly, cells were resuspended in Annexin V-FITC (1 μg/mL), and incubated for 10 min at room temperature in the dark, before PI (1 μg/mL) was added and the cell suspension was examined by flow cytometry (FACSCalibur, BD Pharmingen, USA). To assess the dose effect, several Hcy concentrations were evaluated, including 0, 2.5, 5, 10, 20, and 40 mM.

### Immunofluorescence cell staining

Neuro2a cells were plated on glass coverslips in six-well dishes and cultured in the absence or presence of Hcy (5 mM) for 48 h. Cells were washed with PBS and fixed with 4% paraformaldehyde at room temperature for 20 min, then washed with PBS and permeabilized with 0.3% Triton-X-100 for 45 min. After blocking non-specific staining with 3% bovine serum albumin (BSA) for 30 min, cells were incubated with a GAPDH-specific monoclonal antibody (1:200 dilution, CST, USA) at 4°C overnight. After washing, the coverslips were incubated with a rabbit IgG specific Alexa Fluor 488 conjugated secondary antibody (CST) diluted 1:400 in PBS for 1 h at 37°C. Coverslips were washed three times in PBS, then incubated in DAPI (1:5000) for 10 min. After mounting on slides with glycerine, cells were visualized using a laser confocal scanning microscope (LSM 710, Zeiss, Germany).

### Western blot

Nuclear and cytoplasm proteins were extracted with a commercial kit (Biyuntian, China) according to the manufacturer’s instructions. All procedures were performed on ice or at 4°C. Protein concentration was measured with a bicinchoninic acid (BCA) protein measurement kit (ThermoFisher Scientific, USA). Protein was separated by sodium dodecyl sulfate polyacrylamide gel electrophoresis, transblotted to polyvinylidene difluoride membranes (Millipore, USA) and blocked with 5% non-fat milk in PBS with Tween. The blots were incubated at 4°C overnight with GAPDH-directed monoclonal antibodies (CST; Abcam, USA) at 1:10,000 dilution. Following washing, the membrane was further hybridized with a secondary mouse or rabbit IgG specific antibody (Jackson ImmunoResearch, USA) at 1:500 for 1 h at 37°C and secondary antibody binding was detected using enhanced chemiluminescence (ECL kit, ThermoFisher Scientific). Blots were then stripped and reprobed for the subcellular markers β-actin (dilution 1:1000), β-tubulin (dilution 1:200), and histone deacetylase 1 (HDAC1; dilution 1:200; Santa Cruz Biotechnology, USA), and then secondary antibody binding was detected using enhanced chemiluminescence (ThermoFisher Scientific).

### Immunoprecipitation for the detection of acetylated GAPDH protein levels

Neuro2a cells were cultured in the presence or absence of 5 mM Hcy for 48 h, and then washed with pre-cooled PBS. Phenylmethanesulfonylfluoride-containing NP-40 lysis buffer (Biyuntian) was added and cells were incubated at 4°C with rotation for 30 min, then the supernatants were collected by centrifugation (10,000 g for 10 min). Protein concentration was quantified with BCA and samples were stored at -20°C. Five microliters of acetylated-lysine directed antibody (CST) was added to 500-1000 μg total protein, mixed, and rotated overnight at 4°C. Then, 20 µL of 50% protein A/G-agarose (Santa Cruz Biotechnology) was added, followed by rotation at 4°C for 2-4 h. After 5 min centrifugation at 5,000 g, the supernatant was removed by syringe. Precipitates were washed with NP-40 lysis buffer 3 times, and supernatants were discarded. Beads were re-suspended in 1× sodium dodecyl sulfate loading buffer, denatured at 100°C for 5 min or at 60°C for 20 min. GAPDH was then detected by western blot.

### Statistical analysis

Each experiment was repeated a minimum of three times and results are reported as means±SE. Results were evaluated by one-way analysis of variance, and differences between means were evaluated by the Student-Newman-Keuls method, and considered to be significant when P<0.05.

## Results

### Hcy reduced Neuro2a cell viability

**Figure 1:**
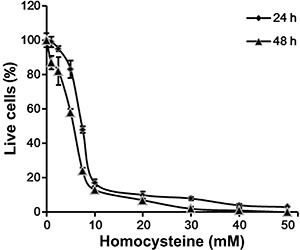
Neuro2a cells were incubated with 10-50 mM homocysteine (Hcy) for 24 or 48 h. Cell viability was assessed with the WST-8 Cell Counting Kit-8 (Dojindo, Japan).

As shown in Figure 1, Neuro2a viability declined in response to Hcy in a dose-dependent manner. The median effective concentration (EC_50_) of Hcy was approximately 5 mM (Figure 1).

### High concentrations of Hcy induced Neuro2a cell apoptosis

**Figure 2:**
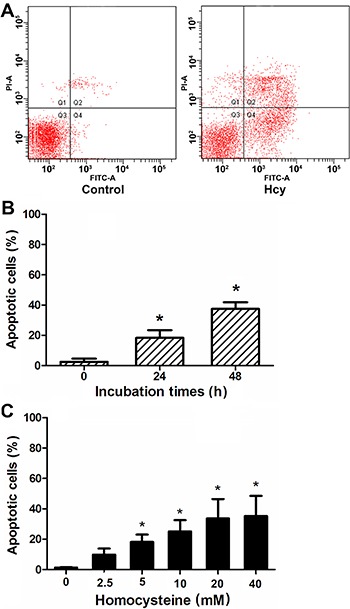
Neuro2a cells were incubated with homocysteine (Hcy) for 24 or 48 h. Apoptotic cell populations were assessed by annexin-V and propidium iodide (PI) staining and flow cytometry. *A*, Representative results of flow cytometry for control and Hcy-treated (5 mM, 48 h) cells. *B*, Apoptotic cell populations in cells treated with 5 mM Hcy for 0, 24, and 48 h. *P<0.05 compared to 0 h (Student-Newman-Keuls test). *C*, Apoptotic cell populations in cells treated 48 h with Hcy. *P<0.05 compared to 0 mM (Student-Newman-Keuls test).

The rate of apoptosis was significantly elevated in cells incubated with Hcy for 24 h (Figure 2B) and it was significantly higher in cells treated with Hcy for 48 h compared to untreated cells, (P<0.05; Figure 2). Hcy-induced apoptosis was dose-dependent, and significantly elevated in cells treated with 5 mM or higher concentrations of Hcy (P<0.05; Figure 2C).

### High concentrations of Hcy induced the nuclear transfer of GAPDH

**Figure 3:**
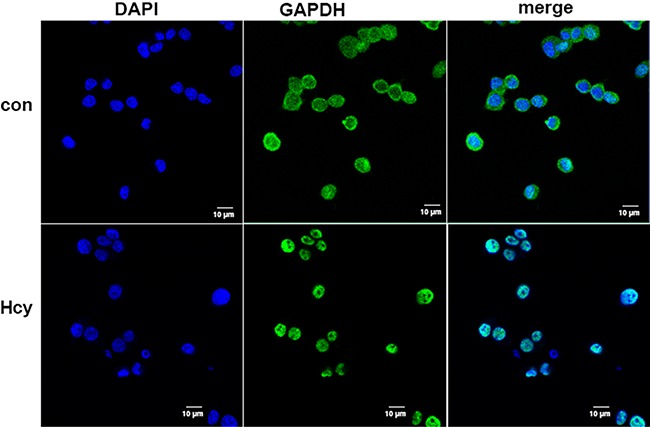
Immunofluorescence of glyceraldehyde 3-phosphate dehydrogenase (GAPDH) cell location before and after homocysteine (Hcy) treatment. Neuro2a cells in the presence of 5 mM Hcy or absence (control [con]) of Hcy for 48 h were then fluorescently labeled with a GAPDH-directed antibody and 4′,6-diamidino-2-phenylindole (DAPI). Representative images of each fluorescent channel and merged images are shown. Scale bar: 10 μm.

**Figure 4:**
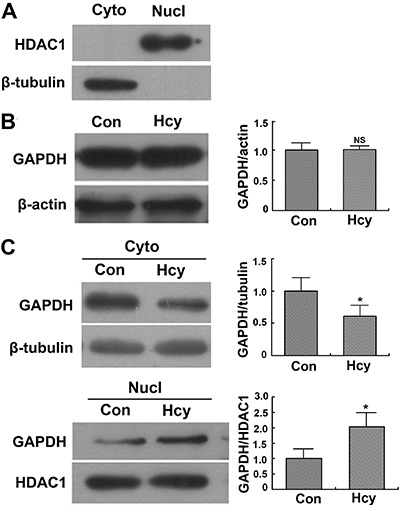
Translocation of glyceraldehyde 3-phosphate dehydrogenase (GAPDH) detected by Western blotting. Cytoplasmic (Cyto) and nucleic (Nucl) proteins were separated from Neuro2a cells incubated in the presence of 5 mM homocysteine (Hcy) or absence (Con) of Hcy for 48 h. The protein levels of GAPDH in subcellular fractions were detected by Western blotting with beta-tubulin as control. *A*, Purification of subcellular fractions was confirmed with the nucleic marker protein histone deacetylase 1 (HDAC1) and cytoplasmic protein marker beta-tubulin. *B*, Total GAPDH protein level. *C*, GAPDH protein levels in cytoplasm and nucleus. *P<0.05 (Student-Newman-Keuls test). NS: not significant.

We investigated the subcellular location of GAPDH in Neuro2a cells after incubation in the presence or absence of Hcy. Confocal fluorescence microscopy revealed that a significantly higher proportion of GAPDH was present in the nucleus of Neuro2a cells incubated with 5 mM Hcy for 48 h than in Neuro2a cells incubated in the absence of Hcy (Figure 3). Western blotting revealed that the total cellular GAPDH content did not differ significantly between cells incubated in the presence or absence of Hcy (P>0.05; Figure 4A). However, the level of GAPDH in the nucleus was significantly higher in Hcy-treated cells than in Hcy-untreated cells (Figure 4B), and the level of GAPDH in the cytoplasm was significantly lower in Hcy-treated cells than in Hcy-untreated cells (Figure 4C).

### High concentrations of Hcy induced acetylation of GAPDH and enhance expression of p300/CBP

**Figure 5:**
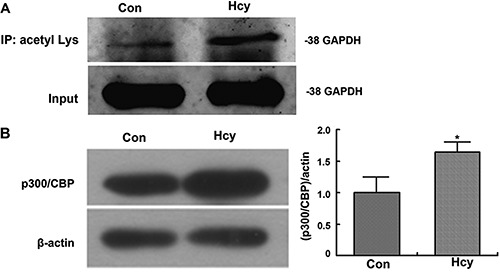
Glyceraldehyde 3-phosphate dehydrogenase (GAPDH) acetylation and expression level of p300/CBP protein. Neuro2a cells were incubated in the presence of 5 mM homocysteine (Hcy) or absence (control [con]) of Hcy for 48 h. *A*, Acetylated proteins in whole cell extracts were immunoprecipitated with acetylated lysine antibody. The GAPDH fractions were then detected by Western blotting. *B*, Protein levels of p300/CBP in whole cell extracts were detected by Western blotting. *P<0.05 (Student-Newman-Keuls test).

Recent studies have shown that nuclear GAPDH mediates cell death through p300/CREB binding protein (CBP). After transfer into the nucleus, GAPDH is acetylated by the acetyltransferase, p300/CBP (27,28). To assess the acetylation of GAPDH in cells incubated with Hcy, we collected acetylated protein by immunoprecipitation with an antibody specific for acetylated lysine, and detected the level of GAPDH by western blot. We found that in Neuro2a cells, GAPDH acetylation increased significantly after incubation with Hcy (Figure 5A). We also observed that the level of acetyltransferase p300 and CBP were significantly higher in cells incubated with Hcy (Figure 5B).

## Discussion

Hcy has been reported to promote the progression of neurodegenerative diseases. However, the mechanism by which Hcy mediates neurotoxicity has not been elucidated. To investigate the mechanism by which Hcy induces neuronal cell death we incubated the mouse neuroblastoma cell line Neuro2a in the presence or absence of Hcy and studied cellular behavior.

We observed that incubation with Hcy significantly reduced the viability of Neuro2a cells, and that Hcy induced Neuro2a apoptosis in a time- and dose-dependent manner. These observations suggest that elucidating the mechanisms involved in Hcy induction of apoptosis might identify potential new target molecules for the treatment of neurodegenerative diseases.

To assess which pathways were activated in Hcy-induced apoptosis, we investigated the pattern of GAPDH distribution and acetylation in Neuro2a exposed to Hcy. GAPDH is also implicated in the mechanism of neuronal apoptosis observed in neurodegenerative diseases (18,20,21,25). In non-apoptotic cells, GAPDH is primarily located in the cytoplasm, whereas in apoptotic cells, GAPDH accumulates in the nucleus (1-3,27). While the total cellular content of GAPDH was not altered in Neuro2a exposed to Hcy, significant translocation of GAPDH from the cytoplasm to the nucleus was observed in response to Hcy. Recent reports suggest that nuclear GAPDH mediates cell death through p300/CBP. After transfer into the nucleus, GAPDH is acetylated by the acetyltransferase p300/CBP, which in turn stimulates the acetylation and catalytic activity of p300/CBP. Consequently, downstream targets of p300/CBP, such as p53, are activated and cause cell death (6,28). To confirm the role of GAPDH in Hcy-induced apoptosis we assessed the level of GAPDH acetylation and found it increased significantly after incubation with Hcy and that the level of acetyltransferase p300 and CBP were significantly higher in cells incubated with Hcy.

These observations suggest that GAPDH nuclear translocation, acetylation, and p300/CBP activity may be involved in the Hcy induction of apoptosis, further highlighting a pathway that could be targeted for the treatment of neurodegenerative diseases.

Previous studies found that translocation of GAPDH could be associated with activation of the nitric oxide (NO)-GAPDH-Siah1-p300/CBP apoptosis pathway. Hara et al. (28) reported that cellular stress increased NO levels in cells, which in turn induced nitrosylation of GAPDH at non-nuclear localization sequences. Thus, modified GAPDH binds to Siah1, an ubiquitin E3 ligase, and is transferred into the cell nucleus via nuclear localization sequences on Siah1. After entering the nucleus, GAPDH is acetylated by p300/CBP, an acetyltransferase, which in turn improves the catalytic activity and acetylation activity of p300/CBP, activating downstream molecules including p53, all of which finally induces apoptosis (27 28-29).

In summary, our observations indicate that Hcy induced Neuro2a cell apoptosis, and the mechanisms of programmed cell death appeared to involve activation of the apoptosis pathway induced by the nuclear translocation of GAPDH. These findings highlight a single signaling pathway involved in neuronal damage mediated by Hcy that may be active in neurodegenerative diseases. However, how Hcy induced the nuclear translocation of GAPDH remains to be determined. Further steps in this pathway will need to be elucidated before we can fully understand how Hcy induces neurotoxicity. Previous reports have implicated alternative pathways in Hcy-mediated neurotoxicity, including activation of N-methyl-D-aspartate (NMDA), non-NMDA, and metabotropic glutamate receptors by Hcy directly, or by Hcy metabolites (reviewed in 30). HHcy has also been reported to induce oxidative stress in neurons as Hcy accumulates and undergoes auto-oxidation (30 31 32 33-34). Nonetheless, these findings do not rule out multiple signaling mechanisms that connect these properties of Hcy. This study highlights novel potential targets for therapies aiming to ameliorate the effects of HHcy and inhibit neurodegeneration.
